# FOXM1 Participates in Trophoblast Migration and Early Trophoblast Invasion: Potential Role in Blastocyst Implantation

**DOI:** 10.3390/ijms25031678

**Published:** 2024-01-30

**Authors:** Reyna Peñailillo, Victoria Velásquez, Stephanie Acuña-Gallardo, Felipe García, Mario Sánchez, Gino Nardocci, Sebastián E. Illanes, Lara J. Monteiro

**Affiliations:** 1 Program in Biology of Reproduction, Center for Biomedical Research and Innovation (CiiB), Universidad de los Andes, Santiago 7620001, Chile; rpenailillo@uandes.cl (R.P.); vpvelasquez@uc.cl (V.V.); sacuna@uandes.cl (S.A.-G.); fagarcia@uandes.cl (F.G.); sillanes@uandes.cl (S.E.I.); 2 IMPACT, Center of Interventional Medicine for Precision and Advanced Cellular Therapy, Santiago 7620001, Chile; gnardocci@uandes.cl; 3 School of Medicine, Faculty of Medicine, Universidad de los Andes, Santiago 7620001, Chile; 4 Program in Neuroscience, Centre for Biomedical Research and Innovation (CiiB), Universidad de los Andes, Santiago 7620001, Chile; mesanchez@uandes.cl; 5 Molecular Biology and Bioinformatics Lab, Program in Molecular Biology and Bioinformatics, Centre for Biomedical Research and Innovation (CiiB), Universidad de los Andes, Santiago 7620001, Chile

**Keywords:** FOXM1, blastocyst implantation, trophoblast invasion and migration

## Abstract

Successful implantation requires coordinated migration and invasion of trophoblast cells into a receptive endometrium. Reduced forkhead box M1 (FOXM1) expression limits trophoblast migration and angiogenesis in choriocarcinoma cell lines, and in a rat model, placental FOXM1 protein expression was significantly upregulated in the early stages of pregnancy compared to term pregnancy. However, the precise role of FOXM1 in implantation events remains unknown. By analyzing mice blastocysts at embryonic day (E3.5), we have demonstrated that FOXM1 is expressed as early as the blastocyst stage, and it is expressed in the trophectoderm of the blastocyst. Since controlled oxygen tension is determinant for achieving normal implantation and placentation and a chronic hypoxic environment leads to shallow trophoblast invasion, we evaluated if FOXM1 expression changes in response to different oxygen tensions in the HTR-8/SVneo first trimester human trophoblast cell line and observed that FOXM1 expression was significantly higher when trophoblast cells were cultured at 3% O_2_, which coincides with oxygen concentrations in the uteroplacental interface at the time of implantation. Conversely, FOXM1 expression diminished in response to 1% O_2_ that resembles a hypoxic environment in utero. Migration and angiogenesis were assessed following FOXM1 knockdown and overexpression at 3% O_2_ and 1% O_2_, respectively, in HTR-8/SVneo cells. FOXM1 overexpression increased transmigration ability and tubule formation. Using a 3D trophoblast invasion model with trophospheres from HTR-8/SVneo cells cultured on a layer of MATRIGEL and of mesenchymal stem cells isolated from menstrual fluid, we observed that trophospheres obtained from 3D trophoblast invasion displayed higher FOXM1 expression compared with pre-invasion trophospheres. Moreover, we have also observed that FOXM1-overexpressing trophospheres increased trophoblast invasion compared with controls. HTR-8/SVneo-FOXM1-depleted cells led to a downregulation of *PLK4*, *VEGF*, and *MMP2* mRNA expression. Our current findings suggest that FOXM1 participates in embryo implantation by contributing to trophoblast migration and early trophoblast invasion, by inducing transcription activation of genes involved in these processes. Maternal-fetal communication is crucial for trophoblast invasion, and maternal stromal cells may induce higher levels of FOXM1 in trophoblast cells.

## 1. Introduction

Embryo implantation is a tightly regulated process by which a competent blastocyst reaches the maternal endometrium at its most receptive phase (window of implantation). This process begins when the outer layer of the blastocyst, the trophectoderm cells, come into contact and attach to the endometrial epithelium (apposition-attachment) [1,2,3]. Shortly thereafter, the trophectoderm undergoes differentiation, giving rise to two different regions: (i) syncytiotrophoblast, a multinucleated outer cell layer that penetrates the endometrial epithelial membrane, allowing the embryo’s implantation into the endometrium; and (ii) mononuclear cytotrophoblast cells that proliferate to form cell columns that further penetrate the endometrial stroma [1,3,4]. Emerging from the tips of these anchoring villi structures are extravillous cytotrophoblast cells (EVT), known for their highly migratory, proliferative, and invasive characteristics [5,6,7].

Trophoblast invasion of the uterus involves attachment of EVT to the extracellular matrix and degradation of the matrix as well as the uterine vasculature (endovascular invasion) to ultimately establish the uteroplacental circulation [3,5]. When this physiological equilibrium is disrupted, implantation failure and subsequent spontaneous abortion may occur [3]. Moreover, shallow trophoblast invasion and impaired uterine vasculature remodeling during the early stages of gestation can also perpetuate during pregnancy and lead to pregnancy complications such as preeclampsia, intrauterine growth restriction (IUGR), and premature birth [7,8,9]. Despite significant progress in reproductive research, efforts are still needed towards a better understanding of the physiological processes initiated during implantation, such as trophoblast invasion and migration, as well as the proteins that might orchestrate them. These two processes are closely related since both require changes in the cell microenvironment and concomitant activation of extracellular proteases [10]. The proteolytic activity of extracellular matrix-degrading metalloproteinases (MMPs) has been largely implicated in the efficiency of trophoblast invasion, specifically in the disruption of the extracellular matrix at the fetal-maternal interface during embryo implantation [11]. 

We have previously demonstrated, in a model of extravillous trophoblast of choriocarcinoma cell lines, that the forkhead box M1 (FOXM1) transcription factor is an important mediator of angiogenesis and migration, which are key processes involved in early placentation [12]. Additionally, we have also showed in a rat model that placental FOXM1 protein expression decreased as gestational age progresses, further indicating a role for FOXM1 in the early stages of pregnancy [12]. Although our previous results are compelling, the precise role of FOXM1 in implantation and whether FOXM1 is expressed in the pre-implantation blastocyst stage remain unknown. FOXM1 plays a crucial role in a plethora of biological processes, by directly regulating the transcription of downstream target genes involved in cell proliferation, cell cycle progression, angiogenesis, migration, invasion, cell differentiation, and DNA damage repair [13,14]. FOXM1 activity is also required in embryonic and fetal development [15,16]. Indeed, during mice embryogenesis, FOXM1 expression is found in the mesenchymal and epithelial cells of the liver, lung, intestine, renal cortex, and urinary tract; yet, its adult expression pattern is restricted to actively proliferating epithelial cells of the intestine, the spermatocytes and spermatids of the testis, the thymus, and colon [17]. Moreover, Foxm1 knockout mice are characterized by an embryonic lethal phenotype due to severe abnormalities in the development of the heart and liver [18]. In this study, we tested the hypothesis that FOXM1 is expressed in the trophectoderm of the blastocyst and participates in embryo implantation by contributing to trophoblast migration and early trophoblast invasion, by inducing transcription activation of genes involved in these processes.

## 2. Results

### 2.1. Foxm1 Is Expressed in the Inner Cell Mass and in the Trophectoderm of Mice Blastocysts

**Figure 1:**
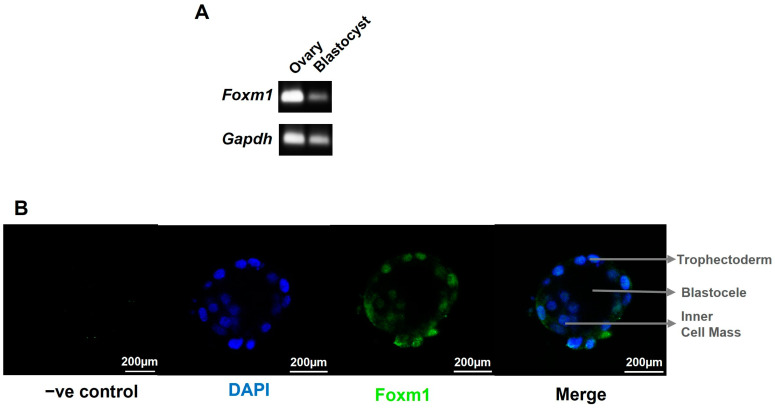
Characterization of Foxm1 in blastocyst. Blastocysts and ovaries were collected from C57BL/6 female mice at embryonic day 3.5 (E3.5). *n* = 4 mice. (**A**) Representative image of 14 blastocysts and 2 ovaries that were used to isolate RNA and amplified by qRT-PCR to evaluate the expression of *Foxm1*. PCR product was run on 1% agarose gel and visualized using a UV transilluminator. Bands correspond to the 101 bp *Foxm1* mRNA template and to the 83 bp *Gapdh* mRNA template that was incorporated as housekeeping gene control. (**B**) Blastocysts were pipetted into drops onto a glass slide and stained for FOXM1 with Alexa Fluor 488 anti-mouse sera (green) and counterstained with DAPI (blue) to visualize the nuclei. Single plane images were acquired with Leica TCS SP8 with a 20× air (N.A. = 0.5) objective. −ve control, negative control: no primary antibody was added. N.A., numerical aperture.

We have previously demonstrated that, in rat placentae, FoxM1 expression decreased as pregnancy progressed from E14.5 to E20.5, suggesting that FOXM1 is important in early placentation events [12]. To further elucidate the role of FOXM1 in the earlier stages of pregnancy, we first sought to indagate whether it is expressed as early as the stage of the pre-implantation blastocyst. Indeed, as seen in Figure 1A, mice embryos in stage E3.5 (early blastocysts) express *Foxm1* mRNA. Mice ovarian tissue was used as the positive control, and a band of *Foxm1* can be appreciated. *Gapdh* was included as the blastocyst housekeeping gene control. Regarding the localization of Foxm1 within the blastocyst, we observed that Foxm1 is expressed not only in the inner cell mass but also in the outer layer of the blastocyst cells (Figure 1B), namely the trophectoderm, an epithelial monolayer of cells, that differentiates into trophoblast cells and directly attaches to and invades the receptive endometrium to establish the placenta. These results strongly suggest that FOXM1 is involved in implantation and early placentation.

### 2.2. FOXM1 Expression Peaks at Time of Implantation in a Human First Trimester Trophoblast Cell Line

**Figure 2:**
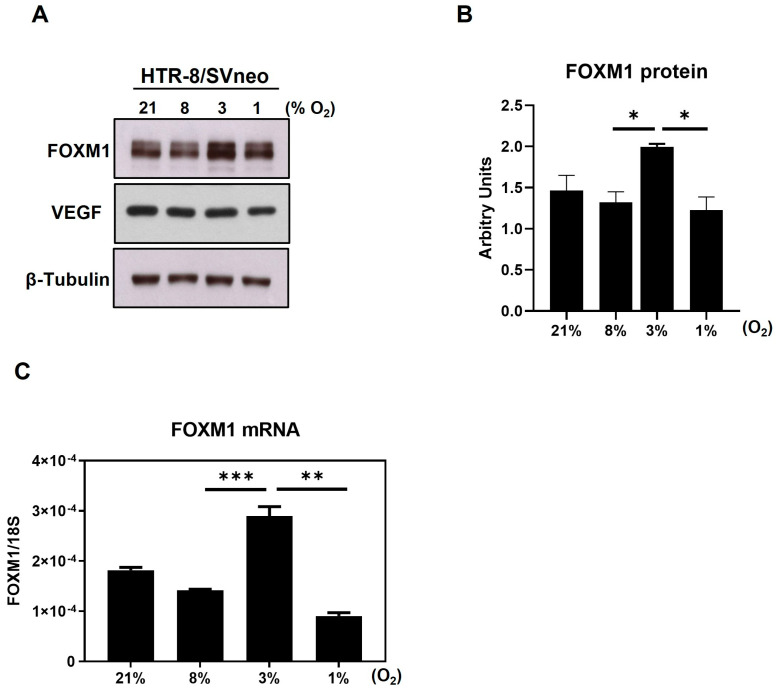
Expression of FOXM1 in the presence of different oxygen percentages. HTR-8/SVneo cells were exposed to 21%, 8%, 3%, and 1% O_2_ for 24 h. (**A**) Cells were then collected and analyzed by western blot to determine the protein expression levels of FOXM1, VEGF, and β-Tubulin (loading control) (representative image of 3 independent experiments is shown). (**B**) FOXM1 band density was quantified and normalized to β-Tubulin. (**C**) qRT-PCR was performed to determine *FOXM1* mRNA transcript levels normalized to *18S* mRNA expression. Results are the mean ± SEM of three independent experiments in duplicate. Statistical analysis was performed using Student’s *t*-tests. * *p* ≤ 0.05, ** *p* ≤ 0.01, *** *p* ≤ 0.001, significant. SEM, standard error of the mean.

To explore whether FOXM1 plays a role in implantation, we analyzed its expression in a cell model of human first trimester trophoblasts, the HTR-8/SVneo cell line. It has been postulated that, in human pregnancy, the oxygen concentration within the uterine surface typically ranges from 2.5 to 5% O_2_ (<20 mmHg) until the eighth week [19]. After the placenta establishes connections with maternal vasculature, that is around 12 weeks, the oxygen concentrations rise until around 8.5% (~60 mmHg). This remains steady until birth [8,19,20,21]. Having that controlled oxygen tension is determinant to achieve normal implantation and placentation, and since a chronic hypoxic environment leads to shallow trophoblast invasion, it is relevant to evaluate if FOXM1 expression changes in response to different oxygen tensions in first trimester human trophoblast cells. Thus, following exposure of HTR-8/SVneo cells to 21%, 8%, 3%, and 1% O_2_, we observed that both FOXM1 protein (Figure 2A) and mRNA expression (Figure 2B) were significantly (*p* = 0.0002) higher when trophoblast cells were cultured at 3% O_2_, which coincides with oxygen concentrations in the uteroplacental interface at the time of implantation. Conversely, we observed FOXM1 expression diminishing in response to 1% O_2_ that resembles a hypoxic environment in utero. VEGF was included as a putative control target of FOXM1 [12,22]. However, we did not observe the same expression trend as with FOXM1.

### 2.3. Overexpression of FOXM1 in HTR-8/SVneo Cell Line Induces Transmigration and Tubule Formation

**Figure 3:**
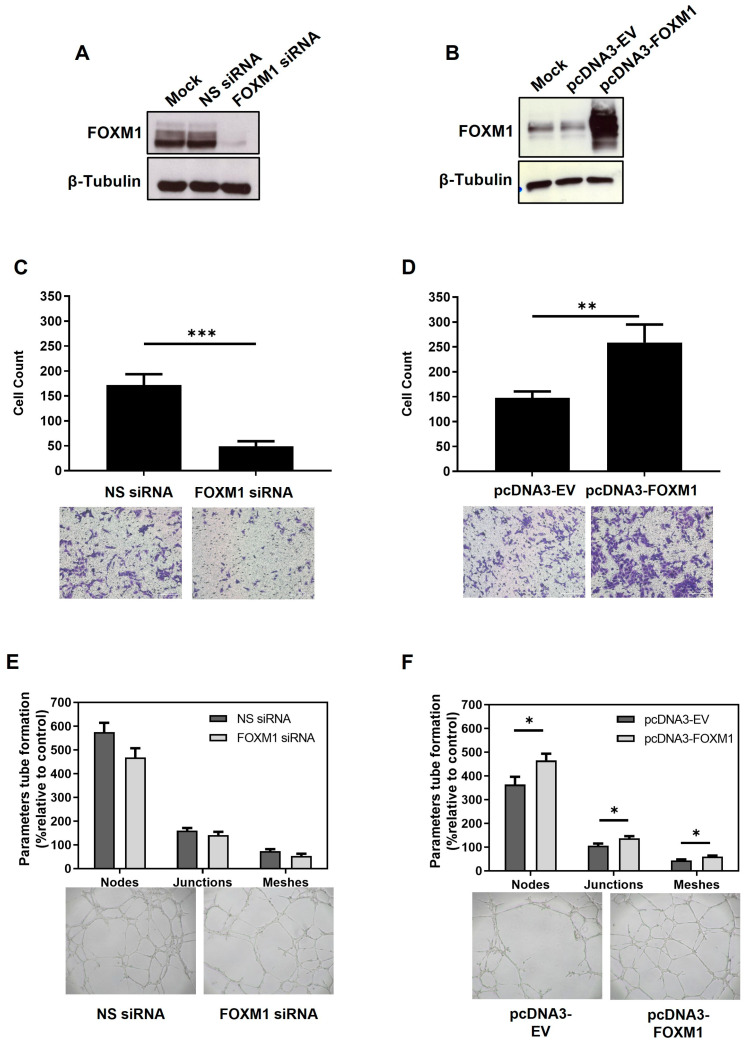
Effects of FOXM1 knockdown and overexpression in the migration and angiogenesis of HTR-8/SVneo cells. (**A**) HTR-8/SVneo cells maintained at 3% O_2_ were transfected with mock (transfection reagent only), non-specific (NS) siRNA or FOXM1 siRNA for 24 h. (**B**) HTR-8/SVneo cells maintained at 1% O_2_ were transfected with mock (transfection reagent only), pcDNA3-empty vector (EV) (control) or pcDNA3-FOXM1 for 24 h. Cells were trypsinized and analyzed for western blotting to confirm: A. FOXM1 silencing and B. FOXM1 overexpression. Fifty thousand remaining cells from A and B were re-seeded in a millicell insert (pore 8 µm) with media with reduced serum (0.5% FBS) and placed on a well of a 48-well plate containing media supplemented with 10% FBS. Transmigration capacity was evaluated after 12 h of incubation, following (**C**) FOXM1 knockdown and following (**D**) FOXM1 overexpression. The represented data are averages of three independent experiments ± SEM. The percentage of migrated cells was calculated: number of cells after/before scraping × 100 (average of the 5 fields). Representative images are shown in the lower panel. Remaining cells from (**A**) and (**B**) were re-seeded onto 96-well plates pre-coated with MATRIGEL to evaluate tubule formation after 6 h of incubation, following (**E**) FOXM1 knockdown and following (**F**) FOXM1 overexpression. Number of nodes, junctions, and meshes were analyzed using ImageJ-Angiogenesis Analyzer-HUVEC Phase Contrast software v1.0.c. Nodes are the intersection of 3 segments, junctions are the combinations of several nodes, and meshes are networks in which nodes are linked together. Data are presented as mean values of three independent experiments ± SEM. Representative images show tube formation networks after 6 h (lower panel). Statistical analysis was conducted using Student’s *t*-tests. * *p* ≤ 0.05; ** *p* ≤ 0.01; *** *p* ≤ 0.001, significant. All images were captured with the Primo Vert microscope (Zeiss, Jena, Germany) with a 4× (N.A. = 0.10, W.D. = 12 mm) objective using the AxioCam ERc5s camera (Zeiss, Jena, Germany). SEM, standard error of the mean. N.A., numerical aperture.

To investigate if FOXM1 is important for peri-implantation events, we performed gain and loss of function assays in the first trimester human trophoblast cells and assessed whether changes in FOXM1 expression influence migration, angiogenesis, and the invasion ability of trophoblast cells. Since FOXM1 expression increased at 3% O_2_ and diminished at 1% O_2_, we knocked down FOXM1 at 3% O_2_ and overexpressed FOXM1 at 1% O_2_. As seen in Figure 3A,B, FOXM1 protein was efficiently silenced and overexpressed, respectively. Following FOXM1 silencing, a significantly lower (*p* = 0.0001) number of migrating cells was observed compared with the non-specific control cells (Figure 3C). However, HTR-8/SVneo cells depleted for FOXM1 only demonstrated a modest but non-significant difference in the tubule network formation compared to those cells transfected with the NS-siRNA control (Figure 3E). Concomitantly, FOXM1 overexpression significantly increased the transmigration ability (Figure 3D, *p* = 0.0001) and tubule formation (Figure 3F; nodes, *p* = 0.0015; junctions, *p* = 0.0008; meshes, *p* = 0.0014) of HTR-8/SVneo cells compared to empty-vector transfection. Nonetheless, we did not observe significant changes in the ability of HTR-8/SVneo cells to invade following overexpression or silencing of FOXM1, as determined by a transwell invasion assay (Supplementary Figure S1). Altogether, these results suggest that FOXM1 is involved in early placentation events, such as transmigration and angiogenesis.

### 2.4. FOXM1 Is Involved in Early Invasion of the Trophoblast

**Figure 4:**
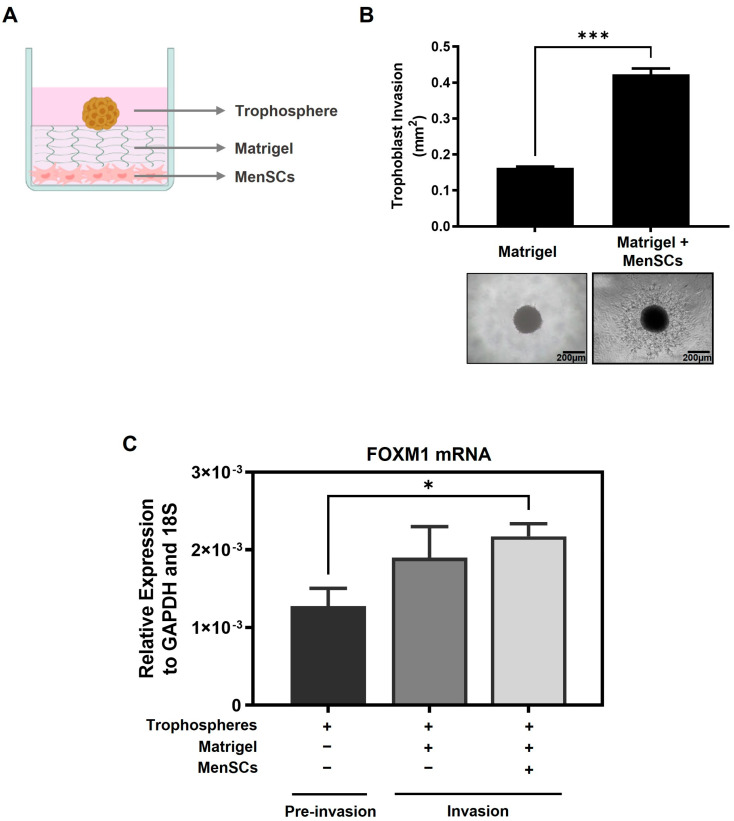
FOXM1 expression is upregulated when trophoblast cells are invading into the matrix. (**A**) Schematic representation of the components of the 3D trophoblast invasion model: MenSCs on the bottom of the plate as a monolayer of endometrial stromal cells, MATRIGEL mimicking the extracellular matrix, and on top is the HTR-8/SVneo trophosphere that is used to mimic the trophectoderm of the blastocyst (image created with BioRender.com). (**B**) HTR-8/SVneo trophospheres were transferred to 8 individual wells of a 96-well plate that contained MATRIGEL alone or a monolayer of MenSCs (*n* = 4) (previously subjected to endometrial mimic) seeded below the MATRIGEL matrix for 72 h. Representative images of trophoblast migration from the trophospheres and invading into the MATRIGEL were captured at day 3 with an Olympus CKX41 microscope and an Axiocam 208 color camera (10× objective lens, N.A. = 0.25, W.D. = 10.5 mm). (**C**) Bars represent: in black: trophospheres immediately after formation, in dark grey: trophospheres in MATRIGEL, and in light grey: trophospheres in MATRIGEL with MenSCs after 3D invasion assay. Trophospheres were collected and recovered from MATRIGEL, and FOXM1 mRNA was analyzed using qRT-PCR and normalized to *GAPDH* and *18S* housekeeping genes. Results are expressed as mean ± SEM of eight trophospheres and *n* = 4 MenSCs. Statistical analysis was conducted using Student’s *t*-tests. * *p* ≤ 0.05; *** *p* ≤ 0.001, significant. MenSCs, mesenchymal stem cells isolated from menstrual fluid; SEM, standard error of the mean. N.A., numerical aperture.

**Figure 5:**
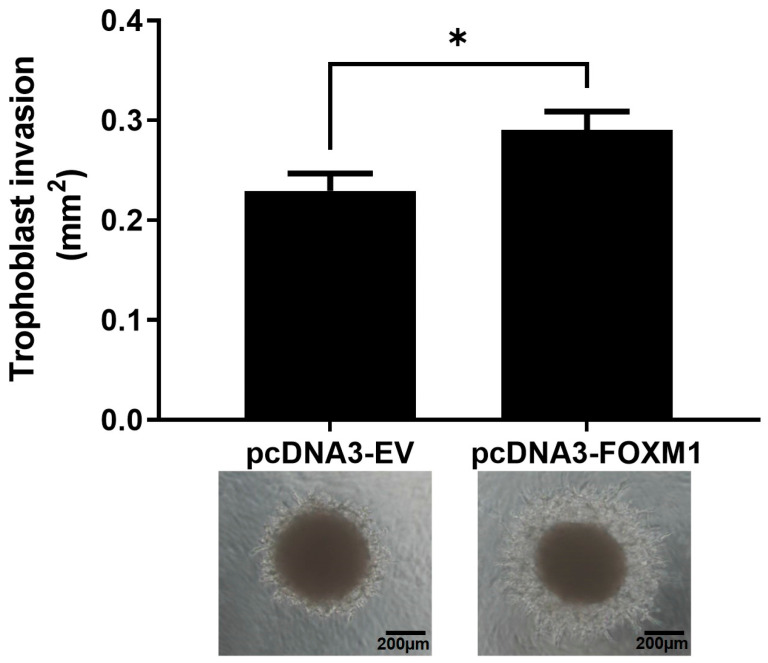
Trophoblast invasion increases in FOXM1-overexpressed trophospheres. HTR-8/SVneo cells were transfected either with pcDNA3-empty vector (EV) (control) or pcDNA3-FOXM1 for 24 h prior to trophosphere generation. Trophospheres were then transferred onto MATRIGEL + MenSCs and trophoblast invasion was evaluated after 48 h. Phase contrast images were captured by the Olympus CKX41 and Axiocam 208 camera (10× objective lens, N.A. = 0.25, W.D. = 10.5). Trophoblast invasion was quantified as area (mm^2^) of trophosphere + invasive trophoblasts (projections that emerge from the trophosphere) by using the ImageJ software v1.54h. Columns are the mean of 2 independent experiments with 6 trophospheres per condition; bars ± SEM. Statistical analysis was conducted using Student’s *t*-tests. * *p* ≤ 0.05, significant. SEM, standard error of the mean. N.A., numerical aperture.

During implantation, trophoblast invasion of the uterine lining depends on the communication between the trophectoderm and the maternal decidua and involves the migration and invasion of the trophoblast cells from out of the blastocyst and through various layers of the endometrium, accompanied by the degradation of the extracellular matrix and stroma [5,10]. In order to understand whether FOXM1 is involved in trophoblast invasion during implantation, we used an in vitro 3D trophoblast invasion model [23,24] that mimics the processes of migration and early invasion of the trophoblast and assessed the expression of FOXM1 in trophoblast cells. This assay consists of generating, in vitro, blastocyst like-structures (trophospheres) and co-culturing them onto a layer of MATRIGEL and mesenchymal stem cells isolated from menstrual fluid (MenSCs), mimicking the extracellular matrix and the endometrial stromal cells, respectively (Figure 4A). To resemble the physiological hormones and O_2_ conditions within the endometrium during the mid-secretory phase of the menstrual cycle, MenSCs were subjected to E2 for 24 h followed by E2 and P4 for an additional 24 h at 5% O_2_ (endometrial mimic), prior to the co-culture with trophospheres. Following 72 h of the co-culture, we observed the formation of projections that penetrated the MATRIGEL in a radial orientation (Figure 4B, top image), which are trophoblast cells that have migrated from the compact blastocyst-like spheroid and invaded the MATRIGEL. In contrast, we hardly observed trophoblast invasion when the trophospheres were cultured only with MATRIGEL, demonstrating that MATRIGEL without MenSCs does not provide the necessary signals to promote trophoblast migration and invasion. Interestingly, we found that FOXM1 transcript levels of the trophospheres that were co-cultured with MenSCs and MATRIGEL were significantly higher compared to trophospheres before 3D invasion assay (Figure 4C, *p* = 0.02), indicating that FOXM1 may be required during early trophoblast invasion of the maternal decidua. Additionally, trophospheres that were generated with pcDNA3-FOXM1 cells exhibited a significant increase (*p* = 0.03) in the area invaded by the trophosphere, compared to those cells transfected with pcDNA3-empty vector control (Figure 5). These results further suggest that FOXM1 plays a role in early invasion of the trophoblast and that this process depends on communication mechanisms between maternal decidual cells and the trophoblast cells of the blastocyst.

### 2.5. FOXM1 Modulates PKL4, VEGF, and MMP2 Transcript Expression in HTR8/SVneo Cells

**Figure 6:**
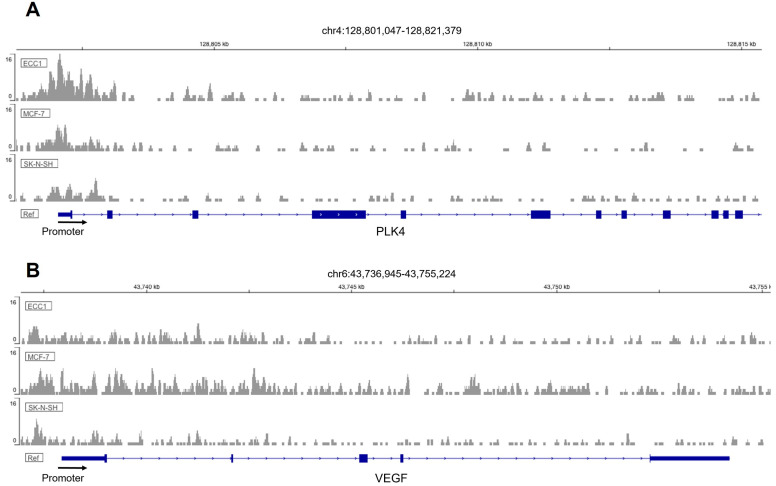
ChIP-Seq enrichment of FOXM1 in 3 different cell lines. Genome binding of FOXM1 transcription factor to (**A**) PLK4 and (**B**) VEGF genes in different cell lines (ECC1, MCF-7, and SK-N-SH). The *x*-axis represents genomic coordinates and the *y*-axis for each track is auto-scaled to the highest peak in each chromosome region shown.

**Figure 7:**
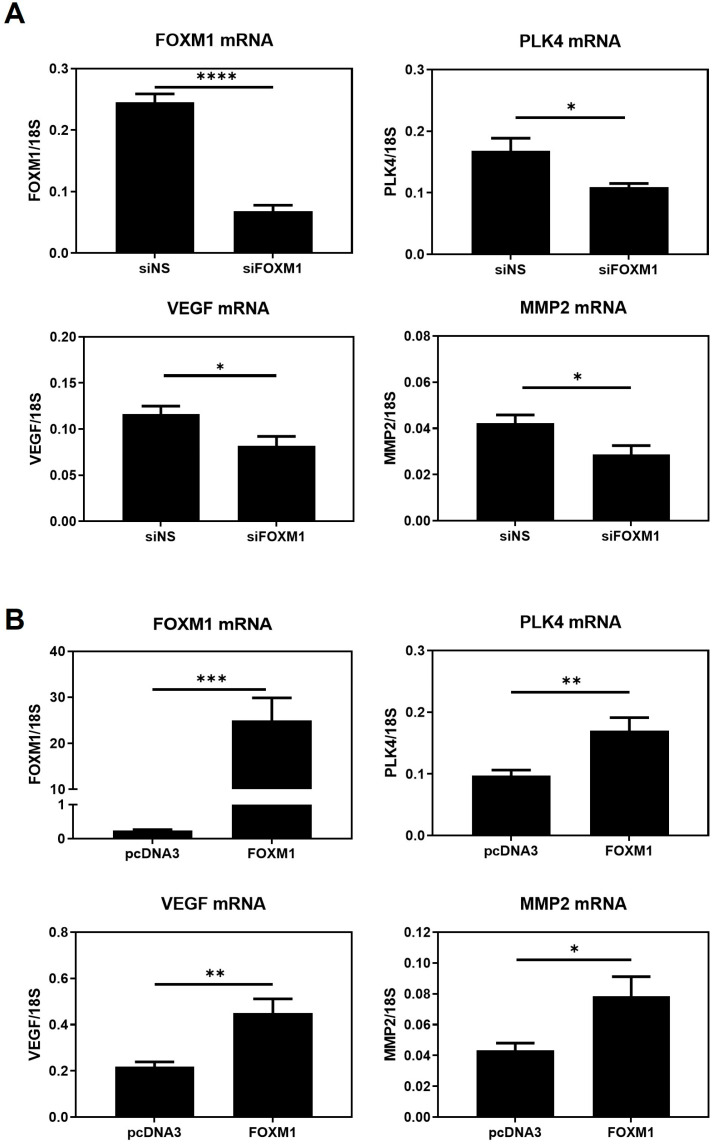
FOXM1 modulates *VEGF*, *PLK4*, and *MMP2* transcript levels in HTR-8/Svneo cells. (**A**) HTR-8/Svneo cells cultured at 3% O_2_ were either transfected with NS siRNA or with FOXM1 siRNA specific pool. (**B**) HTR-8/Svneo cells cultured at 1% O_2_ were transfected with pcDNA3-empty vector (EV) (control) or pcDNA3-FOXM1. Twenty-four hours post-transfection, qRT-PCR analysis was conducted to examine *FOXM1*, *MMP2*, *VEGF*, and *PLK* mRNA expression. All qRT-PCR results were normalized to *18S* mRNA expression. Columns are the mean of 3 independent experiments in duplicate; bars ± SEM. Statistical significance was performed using Student’s *t*-tests. * *p* ≤ 0.05; ** *p* ≤ 0.01; *** *p* ≤ 0.001; **** *p* ≤ 0.0001; significant. SEM, standard error of the mean.

Fox proteins have been reported to bind to the core consensus sequence AAA(C/T)A [25] of their targets´ promoters in order to promote transcription. This consensus sequence is frequently referred to as forkhead responsive element (FHRE). Since FOXM1 may be modulating migration and invasion of trophoblast cells, we seek to identify potential FOXM1 targets whereby it could be mediating these processes. Thus, we analyzed chromatin immunoprecipitation followed by sequencing (ChIP-seq) to investigate the genome-wide chromatin binding mechanisms used by FOXM1. Data for FOXM1 ChIP-seq experiments were obtained from the NCBI GEO database using the accession number GSE32465 [26]. We downloaded three FOXM1 ChIP-seq experiments conducted on ECC1 (human endometrium adenocarcinoma cell line), MCF-7 (human breast adenocarcinoma cell line), and SK-N-SH (human neuroblastoma cell line) cell lines and visualized them using the Interactive Genomic Viewer (IGV). As shown in Figure 6A, FOXM1 binds largely to the proximal promoter regions of *PLK4* and *VEGF* of the different cancer cell lines analyzed (ECC1, MCF-7, and SK-N-SH). To validate these findings in HTR-8/Svneo cells, we evaluated the transcription expression of *VEGF* and *PLK4* following knockdown and overexpression of FOXM1 in HTR-8/Svneo cells (Figure 7A,B). In concordance with the in silico analysis, the depletion of FOXM1 led to a downregulation of *VEGF* (*p* = 0.03) and *PLK4* (*p* = 0.01) mRNA expression, and a significant increase in the transcription levels of these genes was detected following ectopic expression of FOXM1 (*VEGF*, *p* = 0.005; *PLK4*, *p* = 0.009). Since MMP2 has been identified to be secreted by the cytotrophoblast cells that are responsible in digesting the major constituents of the endometrial matrix [27] and has been demonstrated to be a direct target of FOXM1 in retinoblastoma cells [28], we also evaluated the mRNA expression of *MMP2* following gain (*p* = 0.03) and loss (*p* = 0.03) of FOXM1 expression. Interestingly, we observed that MMP2 also followed the same pattern of FOXM1 (Figure 7A,B). Altogether, these results provide evidence that FOXM1 binds to the promotors and directly regulates the transcription of *VEGF*, *MMP2*, and *PLK4* genes, which have important roles in angiogenesis, invasion, and trophoblast differentiation, respectively.

## 3. Discussion

Implantation and early development of the placenta are highly regulated processes that are pivotal for a successful pregnancy. Indeed, there is large body of evidence showing that the impairment of these processes contributes to the etiopathology of pregnancy complications such as preeclampsia, intrauterine growth restriction, and preterm labor. An improved understanding of the molecular mechanisms involved in normal implantation and early trophoblast development will improve the ability of clinicians to understand and treat these pregnancy disorders. Using a model of choriocarcinoma cell lines that exhibits an extravillous trophoblast phenotype, we showed that FOXM1 is an important mediator of key processes involved in early placentation including angiogenesis and migration [12]. Here, we further demonstrate that, in a model of first trimester trophoblast cells, FOXM1 expression significantly increases in response to 3% O_2_, which coincides with the oxygen concentration within the uterine surface at implantation, and 1% oxygen, resembling a hypoxic environment, diminished FOXM1 expression [19]. Moreover, we have observed, for the first time, that FOXM1 is expressed as early as the blastocyst stage in mice embryos and that it is not only is expressed in the inner cell mass but also in the trophectoderm, highlighting the potential role of this protein in the early phases of trophoblast differentiation and blastocyst implantation. This process begins when the trophectoderm cells of the blastocyst attach to the endometrial epithelium, start to migrate out of the blastocyst, and invade the endometrium [1,2,3]. The detection of FOXM1 in the inner cell mass was not surprising since FOXM1 has been shown to be required for embryonic and fetal development [15,16]. Indeed, during embryogenesis, FOXM1 is expressed to ensure correct development of both epithelial and mesenchymal tissues [17]. Additionally, FOXM1 has been demonstrated to be essential for embryonic development of mice pulmonary vasculature [18].

In the cancer scenario, it has been demonstrated that FOXM1 induces tumor proliferation, migration, invasion, and angiogenesis [14], which are processes shared by trophoblast cells during the peri-implantation phase. Accordingly, we have also demonstrated that FOXM1 is necessary for the migration and angiogenesis of the human HTR8/Svneo first trimester trophoblast cell line while not influencing its proliferative ability (Supplementary Figure S2). Moreover, neither silencing nor overexpression of FOXM1 led to statistically significant effects on the ability of HTR8/Svneo cells to invade MATRIGEL, as assessed by transwell invasion assays (Supplementary Figure S1).

Successful implantation requires effective maternal-embryonic communication [2,3,4,27]. Using an in vitro 3D trophoblast invasion model, we have confirmed that trophoblast cells were only able to migrate from the trophosphere and invade through the MATRIGEL in the presence of MenSCs of endometrial origin, which was pre-treated with the endometrial mimic, suggesting that MenSCs secrete important factors that trigger the motility and invasion of the trophoblast cells, as described previously [10,23,24]. More importantly, FOXM1 mRNA expression was significantly higher in those trophoblast cells that invaded greater into the MATRIGEL, i.e., the trophospheres that were co-cultured with MATRIGEL and MenSCs compared to pre-invasion trophospheres. Together, these results suggest that trophoblast invasion strongly relies on maternal-fetal communication and that maternal decidual cells may induce trophoblast cells to express higher levels of FOXM1 that subsequently activate genes involved in trophoblast migration and invasion. Mitochondria or extracellular vesicle transfer could be studied as potential communication mechanisms. To further validate that FOXM1 is indeed required for trophoblast invasion, we demonstrated that FOXM1-overexpressed trophospheres exhibited a significant increase in the area invaded by the trophosphere, compared to those cells transfected with the pcDNA3-empty vector control. It is worth mentioning that spheroid formation from HTR-8/Svneo morphologically resembles a blastocyst and expresses higher stemness markers, NANOG and SOX2, and lower CDH1 epithelial marker, in comparison with its adherent counterpart [23].

As a transcription factor, it is plausible that FOXM1 exerts its effects by directly binding the promoters and inducing the transcription of genes involved in trophoblast differentiation, migration, invasion, and angiogenesis. Thus, we propose that *PLK4*, *VEGF*, and *MMP2* are FOXM1 downstream targets involved in these processes. Invasion and migration are closely related mechanisms that involve the ability of the cells to move in response to a stimulus and to advance through the extracellular matrix within a tissue by activating extracellular proteases [29]. The proteolytic activity of extracellular matrix-degrading metalloproteinases (MMPs) has been largely implicated in the efficiency of trophoblast invasion, specifically in disrupting the extracellular matrix at the fetal-maternal interface during embryo implantation [11]. Specifically, MMP2 has been identified to be secreted by the cytotrophoblast cells that are responsible in digesting the major constituents of the endometrial matrix [27]. Indeed, MMP2 can be directly regulated by FOXM1 in human retinoblastoma Y-79 cells [28]. Consistently, a large-scale microarray study of the transcriptome of the rat placenta throughout mid-late gestation also reveals the concomitant expression of FOXM1 and angiogenic genes, including VEGFA, MMP14, Caveolin-1, and Angiopoietin-4 [30]. Moreover, VEGF has also been demonstrated to be a direct target of FOXM1 in breast cancer cells [22]. Polo-like kinase four (PLK4) is a member of the serine/threonine kinase family that plays an important role in cell cycle regulation, by participating in centriole duplication and maintaining mitotic accuracy in normal cells. Its deregulation, henceforth, has been associated with a prominent role in cancer and metastasis [31,32]. PLK4 has also been demonstrated to be involved in the differentiation of trophoblast stem cells into trophoblast giant cells during placental development [31,33]. This evidence, together with the ChIP-Seq data, indicates that *VEFG*, *PLK4*, and *MMP2* are potential downstream targets of FOXM1, through which it promotes trophoblast differentiation, migration, invasion, and angiogenesis during early placentation. To support this conjecture, we have also shown that the mRNA expression of these genes is significantly downregulated following silencing of FOXM1 and significantly upregulated following FOXM1 overexpression in HTR-8/Svneo cells. Further studies using ChIP are required to validate that *VEFG*, *PLK4*, and *MMP2* are downstream targets of FOXM1 in HTR-8/Svneo cells.

Our results demonstrate, for the first time, that FOXM1 is present in the trophectoderm of mice blastocysts. By using a 3D in vitro model of implantation, we have confirmed that maternal-fetal communication is crucial for trophoblast invasion and that maternal stromal cells may induce trophoblast cells to express higher levels of FOXM1, which subsequently activates the transcription of its downstream targets genes that are important for successful implantation: *PLK4*, *VEGF*, and *MMP2*.

## 4. Materials and Methods

### 4.1. Cell Culture

The human HTR8/Svneo first trimester trophoblast cell line was purchased from the American Type Culture Collection (CRL-3271; Lot #70016636, ATCC, Manassas, VA, USA) and cultured in RPMI-1640 medium (GE Healthcare, Piscataway, NJ, USA), supplemented with 10% heat-inactivated fetal bovine serum (FBS), 1 mmol/L sodium pyruvate, and 1% penicillin/streptomycin (P/S) (all Gibco, ThermoFisher, Waltham, MA, USA). Cells were maintained at 37 °C in a humidified incubator with 5% CO_2_. For hypoxia experiments, cells were seeded in the appropriate cell culture plastic wells and maintained at 21%, 8%, 3%, or 1% O_2_ for 24 h to 72 h prior to experiments. Hypoxic chamber C-474 equipped with Pro-Ox 110 oxygen controlling regulator (BioSpherix, Parish, NY, USA) was used.

### 4.2. Embryo Collection

C57BL/6 mice were purchased from Universidad de Chile. Animals were maintained in the animal facility of Universidad de los Andes at 25 °C and 12 h light:dark cycling and fed standard chow and water ad libitum. Protocols were conducted in agreement with the National Research Council (NRC) publication Guide for the Care and Use of Laboratory Animals (eighth edition, The National Academies Press, Washington, DC, USA). The animal study was reviewed and approved by the Ethical Scientific Committees of Universidad de los Andes, Santiago, Chile. For blastocyst collection, female mice were superovulated by intraperitoneal injections with 5 IU of pregnant mares’ serum gonadotropin (PMSG) (Novormon, Buenos Aires, Argentina) and 5 IU of human chorionic gonadotropin (HCG) (Sigma-Aldrich, St Louis, MO, USA) 48 h later. Immediately after HCG injection, female mice were mated 1:2 to male mice of the same strain. Mating was evaluated by inspection of the vaginal plug on the following day. The presence of sperm plug was considered as embryonic day 0.5 (E0.5) of pregnancy. Pregnant females were euthanized at E3.5 and subsequently, blastocysts were obtained. The procedure involved initial anesthesia with ketamine:xylazine, followed by euthanasia by cervical dislocation. Embryos were collected by flushing the uterus with M2 medium (Sigma-Aldrich, St Louis, MO, USA). Then, freshly harvested embryos were washed once in M2, followed by three washes with M2 medium containing polyvinyl pyrrolidone (PVP; 6 mg/mL, H6PVP Sigma-Aldrich, USA) and three washes with 1X phosphate-buffered saline (PBS) (Cytiva, Logan, UT, USA). Embryos and oocytes with signs of disintegration were classified as degenerated embryos or oocytes. At least 14 embryos at the blastocyst stage were used for qRT-PCR. The blastocysts were transferred to a tube with a minimal volume (1-2 µL) of PBS, snap-frozen in liquid nitrogen, and stored at −80 °C. For blastocyst differential labelling of cell lineages by immunofluorescence, embryos were washed and used immediately.

### 4.3. Immunofluorescent Staining

Immunofluorescence staining of the blastocyst was carried out as described previously [34]. After embryo collection, blastocysts (E3.5) were washed three times in M2 medium, and the *zona pellucida* was removed using acidified Tyrodes solution (Sigma-Aldrich, USA), at pH 2.3 at 37 °C for 30–60 s, followed by extensive washing in M2. Blastocysts were fixed with 4% PFA (ThermoFisher, Rockford, IL, USA) in PBS for 15 min at room temperature. The fixative was removed, and the embryos were washed three times with 1X PBS and then permeabilized with 0.2% Triton X-100 (Sigma-Aldrich, USA) in 1X PBS for 10 min at room temperature. Embryos were washed again three times with PBS containing 0.1% (*v*/*v*) Tween 20 (Winkler, Santiago, Chile) (PBST), followed by blocking with 1% BSA (Rockland, Philadelphia, PA, USA) and 5% goat serum G9023 (Sigma-Aldrich, St. Louis, MO, USA) in PBST for 1 h at room temperature. FOXM1 G-5 (Santa Cruz Biotechnology, Santa Cruz, CA, USA) primary antibody in PBST, 1% BSA, 1% goat serum, and glycine at 0.05 M (Winkler, Chile) were added overnight at 4 °C. The next day, the embryos were washed three times with PBST with 1% BSA, followed by secondary antibody Alexa Fluor 488 anti-mouse (ThermoFisher, Eugene, OR, USA) incubation for 1 h at room temperature in a dark environment. Final PBST with BSA washes were performed. A drop of Fluoromount-G (Invitrogen, Carlsbad CA, USA) mounting medium with 4′,6-diamidino-2-phenylindole (DAPI) (Vector Laboratories, Burlingame, CA, USA) was added to each drop of blastocysts before addition of a coverslip and sealing with clear nail varnish. After drying, slides were stored at 4 °C. Images were collected with a Leica TCS SP8 confocal microscope (Leica Microsystems, Mannhein, Germany) with 20× objective (N.A. 0.5) using LAS-X software v3.0 (Leica Microsystems, Mannhein, Germany).

### 4.4. Western Blot Analysis

Cells were harvested by trypsinization and whole cell lysates prepared as described previously [35]. Primary antibodies, FOXM1 D12D5 (Cell Signaling, Danvers, MA, USA), β-Tubulin H-235 (Santa Cruz Biotechnology, USA), and VEFG-A ab46154 (Abcam, Cambridge, MA, USA), were detected using horseradish peroxidase-linked anti-rabbit or anti-mouse conjugates as appropriate (KPL, Gaithersburg, MD, USA), and proteins were visualized using enhanced chemiluminescence (ECL) Pierce ECL Western Blotting Substrate detection system (ThermoFisher, USA) with X-ray films. The obtained images were analyzed using ImageJ software v1.54h (National Institutes of Health, Bethesda, MD, USA). All samples were normalized for protein loading using β-tubulin.

### 4.5. RNA Isolation and Quantitative Real-Time PCR

Total RNA was extracted from HTR8/SVneo cells with the TRIzol Reagent (Ambion, Carlsbad CA, USA) according to the manufacturer’s protocols. RNA from the pooled blastocyst (*n* = 14) was isolated using PureLink RNA Micro Kit (Invitrogen, Carlsbad CA, USA) according to the manufacturer’s protocols. RNA concentration and quality were evaluated using the NanoDrop One spectrophotometer (ThermoFisher, Madison WI, USA). Before cDNA synthesis, total RNA was treated with DNase I (Invitrogen, Carlsbad CA, USA). Complementary DNA generated by SuperScript II Reverse Transcription System (Invitrogen, Carlsbad CA, USA), according to the manufacturer’s instructions, was analyzed using quantitative real-time PCR (qRT-PCR) in the Stratagene Mx3000P system (Agilent Technologies, Santa Clara, CA, USA), using Brilliant III SYBR Green qPCR Master Mix (Agilent Technologies, USA). The qRT-PCR was set to 95 °C for 10 min for enzyme activation, followed by 35 cycles of denaturation and primer annealing/extension consisting of 95 °C for 15 s, 60 °C for 15 s, and 72 °C for 15 s, respectively. After the PCR runs, a dissociation curve was generated to confirm the absence of nonspecific amplification. Transcription levels were quantified using the 2^−ΔΔCT^ method. 18S ribosomal RNA or GAPDH housekeeping genes were used to normalize input complementary DNA. The following gene-specific primers were used for mouse: Foxm1-sense: 5′-GGACATCTACACTTGGATTGAGG-3′ and Foxm1-antisense: 5′-TGTCATGGAGAGAAAGGTTGTG-3′; Gapdh-sense: 5′-AGTGGCAAAGTGGAGATT-3′ and Gapdh-antisense 5′-GTGGAGTCATACTGGAACA-3′; and for human HTR8/SVneo cells: FOXM1-sense: 5′-TGCAGCTAGGGATGTGAATCTTC-3′ and FOXM1-antisense: 5′-GGAGCCCAGTCCATCAGAACT-3′; VEGF-sense: 5′-TATGCGGATCAAACCTCACC-3′ and VEGF-antisense: 5′-CTTGTCTTGCTCTATCTTTCTTTGG-3′; MMP2-sense: 5′-TGTGACGCCACGTGACAAG-3′ and MMP2-antisense: 5′-CCAGTATTCATTCCCTGCAAAGA-3′; PLK-4-sense: 5′-GATAGACCACCCTCACCTACT-3′ and PLK-4-antisense: 5′-CTGTACAAACCTGGAAGCATATTG-3′; 18S-sense: 5′-GCCGCTAGAGGTGAAATTCTTGGA-3′ and 18S-antisense: 5′-ATCGCCAGTCGGCATCGTTTAT-3′; GAPDH-sense: GTCAGGGTCTCTCTCTTCCT and GAPDH-antisense: GCTCTCCTCTGACTTGAACA.

### 4.6. Transient Gene Silencing and Overexpression

For gene silencing, HTR8/SVneo cells were transiently transfected with ON-TARGETplus SMARTpool siRNA (Dharmacon ThermoFisher, Lafayette, CO, USA), using Oligofectamine (Invitrogen, Carlsbad, CA, USA) and Opti-MEM (Gibco, ThermoFisher, Waltham, MA, USA) according to the manufacturer’s instructions. siRNA FOXM1 (L-009762-00-0005) and the non-specific (NS) control siRNA (D-001810-10-05) SMARTpools were used. RNA interference experiments were carried out at 3% O_2_. For gene overexpression, HTR8/SVneo cells were transfected with either the pcDNA3-empty vector or pcDNA3-FOXM1 [36] using FuGENE 6 Transfection Reagent (Promega, Madison, WI, USA) in a 3:1 ratio (μL of FuGENE: μg of DNA) according to the manufacturer’s recommendations. The overexpression experiments were performed at 1% O_2_.

### 4.7. Transmigration Assay

HTR8/SVneo cells transiently transfected with either FOXM1 siRNA or control NS siRNA, or pcDNA3-FOXM1 or empty vector EV-pcDNA3, under different oxygen tensions were used to examine cell migration in vitro. Following 24 h of transfection, 50,000 HTR-8/SVneo cells were seeded in a millicell insert (pore 8 µm, 12 mm, Millipore, Billerica, MA, USA) with 400 µL RPMI with reduced serum (0.5% FBS). Inserts were placed on 500 µL of RPMI supplemented with 10% FBS in 48-well plates. Transmigration capacity was evaluated following 12 h incubation either at 3 or 1% O_2_. Briefly, the insert was washed with 1X PBS, fixed with cold methanol for 2 min, and stained with 0.5% crystal violet (Winkler, Santiago, Chile). Cells inside the inserts were scraped with cotton swabs moistened with 1X PBS to ensure that only migrated cells were analyzed. Five fields were captured for each insert at 4× objective magnification (N.A. = 0.10, W.D. = 10.5 mm) before and after scraping under an inverted microscope (Primo Vert, Zeiss, Jena, Germany), using the AxioCam ERc5s camera (Zeiss, Jena, Germany). Images were analyzed with AxioVision Rel analysis software v4.8 (Zeiss, Jena, Germany). The percentage of migrated cells was calculated as follows: number of cells after/before scraping × 100 (average of the 5 fields). The experiments were performed in duplicate.

### 4.8. MATRIGEL-Based Tube Forming Assay

HTR8/SVneo cells transiently transfected with either FOXM1 siRNA or control NS siRNA, or pcDNA3-FOXM1 or empty vector EV-pcDNA3, under different oxygen tensions were resuspended in the RPMI medium with 2% FBS or in the endothelial growth media-2 (EGM-2, Bullet Kit, Lonza, Verviers, Belgium) (positive control) and seeded in triplicate in 96-well plates pre-coated with 70 μL growth factor reduced phenol-red free MATRIGEL matrix (Corning Life Sciences, Union City, CA, USA). Cells were incubated at 37 °C and under 3 or 1% O_2_ (accordingly) for 6 h and tube-like structures were examined with a phase-contrast microscope Primo Vert (Zeiss, Jena, Germany). One image per well was captured using an AxioCam ERc5s camera (Zeiss, Jena, Germany). Number of nodes, junctions, and meshes were analyzed using ImageJ-Angiogenesis Analyzer-HUVEC Phase Contrast software v1.0.c.

### 4.9. Trophosphere Formation

Trophoblast spheres (Trophospheres) are blastocyst like-structures formed in vitro using adherent HTR8/SVneo cells, as described previously [23]. In brief, 20,000 HTR8/SVneo cells were suspended in 200 μL of supplemented RPMI media and placed into each well of an ultra-low attachment 96-well plate (Costar, Kennebunk, ME, USA). Following centrifugation at 300× *g* for 5 min, cells were incubated for 72 h at 37 °C in a humidified atmosphere hypoxia chamber with 5% O_2_ and 5% CO_2_. The trophospheres were washed with 1X PBS and used for 3D invasion assays. The differential cellular characteristics of trophoblast cells as a 3D model or monolayer were previously described in detail [23].

### 4.10. 3D Trophoblast Invasion Assay

To mimic the structure of the endometrium, mesenchymal stem cells isolated from menstrual fluid of healthy donors, who had not used hormonal contraceptives for at least three months, were isolated and cultured as described in [23,24]. All experiments were performed using MenSCs at early passages (P) P3 to P7 (*n* = 4). Menstrual fluid was self-collected by consenting donors following their written informed consent to participate in this study according to a protocol reviewed and approved by the Ethical Scientific Committee of the Universidad de los Andes, Santiago, Chile. To achieve the 3D invasion assay [23], growth factor reduced phenol-red free MATRIGEL (Corning Life Sciences, Union City, CA, USA) was mixed with Dulbecco’s Modified Eagle Medium (DMEM) without phenol red (Mediatech Inc., Manassas, VA, USA) containing 10% charcoal-stripped FBS and 1% P/S in a 1:1 ratio and added onto 3000 MenSCs that had been previously seeded in a 96-well plate and subjected to 213 pg/mL 17β-estradiol (E2) for 24 h, followed by 146 pg/mL E2 and 11 ng/mL progesterone (P4) for an additional 24 h at 5% O_2_ (endometrial mimic), in order to resemble the physiological hormonal and O_2_ conditions within the endometrium, throughout the menstrual cycle [23]. The plate was incubated for 30 min at 37 °C to allow the MATRIGEL to solidify. A single trophosphere was placed on each well containing MenSCs and MATRIGEL, and 150 μL of endometrial mimic media were added to embed the sphere. Plates were incubated at 37 °C in a humidified atmosphere hypoxia chamber with 5% O_2_ and 5% CO_2_. Trophosphere invasion was evaluated after 72 h. Phase contrast images were captured by the contrast microscope Olympus CKX41 and Axiocam 208 camara (10× objective lens, N.A. = 0.25, W.D. = 10.5 mm) and AxioVision Rel software v4.8 (Zeiss, Jena, Germany). The invasion level (area) was quantified by using ImageJ software v1.51j8, and the trophospheres were collected for qRT-PCR assay. For mRNA analysis of the trophospheres following 3D invasion assay, the trophospheres were recovered from MATRIGEL and incubated with 300 μL of Cell Recovery Solution (Corning Life Sciences, Bedford, MA, USA) for 15 min on ice, followed by centrifugation at 400× *g* for 5 min at 4 °C. The supernatant was removed and the trophospheres were washed with PBS, followed with centrifugation at 350× *g* for 2 min at 4 °C. PBS was removed and the trophospheres were lysed with TRIzol for RNA extraction.

### 4.11. Statistical Analyses

Statistical comparisons between two means were performed using Student’s *t*-tests. Results were deemed statistically significant when *p* ≤ 0.05. Data normality was tested using the D’Agostino-Pearson test. For analysis of 2 groups, unpaired *t*-test was used. Graphing and statistical analyses were performed using GraphPad Prism 9.0.

## Data Availability

Data are contained within the article and Supplementary Materials.

## Supplementary Materials

The following supporting information can be downloaded at: https://www.mdpi.com/article/10.3390/ijms25031678/s1.
