# Supplementary material for: FOXM1 Participates in Trophoblast Migration and Early Trophoblast Invasion: Potential Role in Blastocyst Implantation
Source: Int J Mol Sci. 2024 Jan 30;25(3):1678. doi: 10.3390/ijms25031678 (PMC10855960; doi:10.3390/ijms25031678)
Supplement: Supplementary file 1 [file ijms-25-01678-s001.zip › ijms-2825872-supplementary.pdf]

Supplementary Material

FOXM1 participates in trophoblast migration and early trophoblast invasion: a potential role for blastocyst implantation

R Peñaililloᵗ, V Velásquezᵗ, S Acuña-Gallardo, F Garcia, M Sánchez, G Nardocci, SE Illanes, LJ Monteiro*

ᵗThe authors consider that the first two authors should be regarded as joint First Authors

* **Correspondence:** Lara J. Monteiro: lmonteiro@uandes.cl

Supplementary Figures

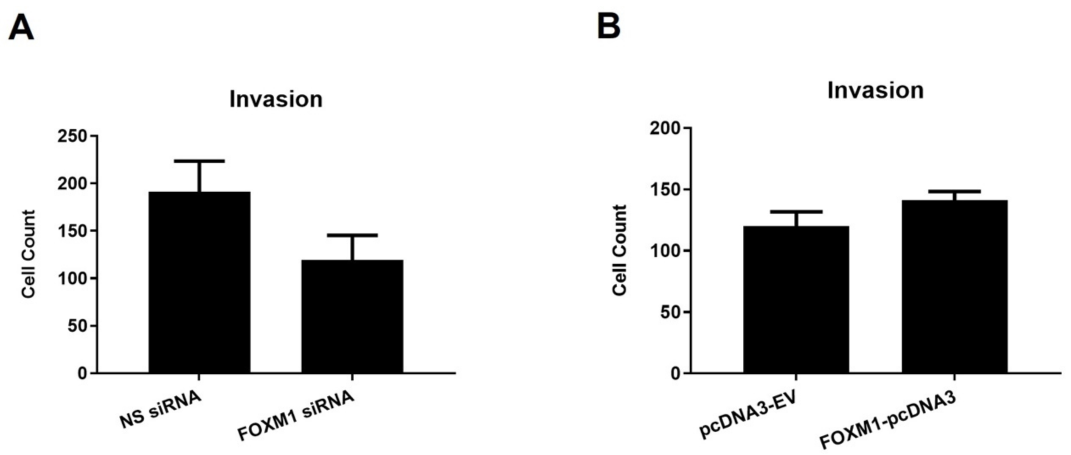

Figure S1- Silencing or overexpression of FOXM1 in HTR-8/SVneo cells did not affect trophoblast proliferation. A. HTR-8/SVneo cells cultured at 3% O_2 were either transfected with NS siRNA or with FOXM1 siRNA specific pool. B. HTR-8/SVneo cells were transfected with pcDNA3-Empty vector (EV) (control), or pcDNA3-FOXM1. 24 h post-transfection 2000 cells were re-seeded in 96 well plates. Cell proliferation and viability were determined at 24, 48 and 72 hours by the sulforhodamine B assay Results are the means ± SEM of 2 independent experiments with 8 replicates per condition. SEM, standard error of the mean.

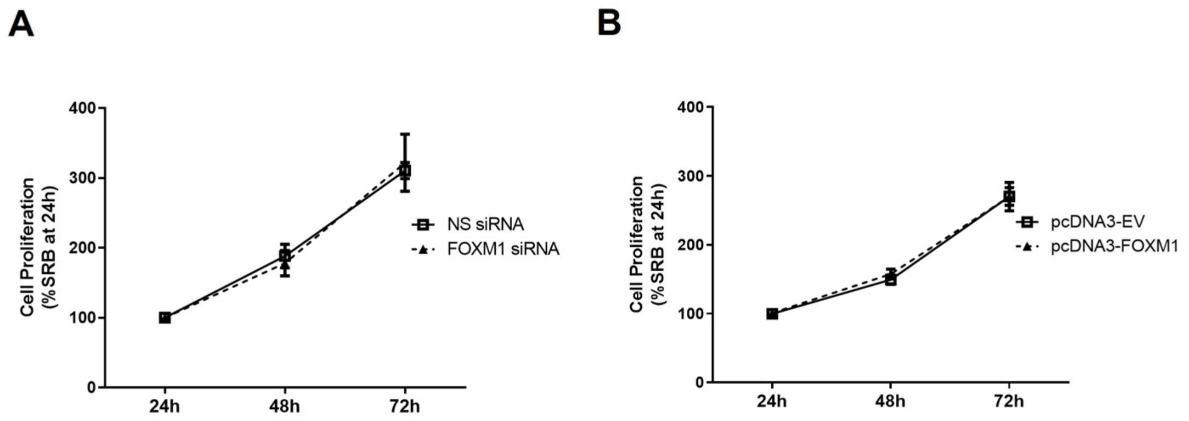

Figure S2- Silencing or overexpression of FOXM1 in HTR-8/SVneo cells did not affect trophoblast invasion in a Boyden chamber precoated with matrigel. A. HTR-8/SVneo cells cultured at 3% O_2 were either transfected with NS siRNA or with FOXM1 siRNA specific pool. B. HTR-8/SVneo cells cultured at 1% O_2 were transfected with pcDNA3-Empty vector (EV) (control), or pcDNA3-FOXM1. 24 h post-transfection 60.000 cells were re-seeded with RPMI 0.5% FBS media into a precoated Boyden chamber (Millicell) with matrigel. The bottom chamber was filled with RPMI 10% FBS media. Cells were incubated for 24 h following. Cells that were able to degrade the matrigel layer and migrate the Boyden chamber membrane were fixed with 4% PFA, permeabilized with methanol and stained with the 0.2% Crystal Violet in 5% ethanol. Five fields were captured for each insert at 20X using the Primo Vert inverted microscope with the AxioCam ERc5s camera. Cell numbers were examined by counting the number of stained cells with the ImageJ software. Results are the means + SEM of 3 independent experiments with 2 replicates per condition. SEM, standard error of the mean.
